# Cases and Observations

**Published:** 1854-12

**Authors:** W. H. Cobb

**Affiliations:** Glasgow, Mo.


### ART. III.—CASES AND OBSERVATIONS.

BY W. H. COBB, M. D., OF GLASGOW, MO.

### *Nitrate of Silver in Diseases of the Cervix Uteri.*

IN common with most practitioners, I have experienced much difficulty in introducing the common Nitrate of Silver within the uterine neck, the caustic being too brittle to render such a proceeding entirely free from risk. All practitioners, who have had much experience in treating diseases of the cervico-uterine neck, must hence feel the need of some combination or modification of the caustic, which rendering it less friable, should divest its introduction of the danger of its breaking off, while it preserved the properties of the nitrate. With a view to obviating this difficulty, Dr. VAUGHAN and myself instituted a series of experiments, combining the nitrate with gum-arabic and other adhesive powders. The result of these manipulations was that by mixing equal parts of powdered caustic and gum-arabic, we formed a compouud at once flexible, less fragile, and capable of producing the usual effects of the original nitrate. They were thoroughly incorporated together with rain-water, rolled into cylinders of the proper dimensions and protected from the action of the light in the same manner as the common caustic. While conducting these experiments Dr. VAUGHAN accidentally brought a portion of the compound thus formed in contact with the blaze of a candle and was surprised to find that it burned with a rapid combustion without sparks, leaving a greyish-white residum. The residum was found upon examination to be metallic silver in a state of minute subdivision, and when freed from impurities by washing with distilled water, might be used by the dentist in forming amalgams, or reconverted into the nitrate. It also occurred to us upon examining the mode of combustion, that the nitrate, thus modified, might subserve the purpose of a moxa superseding the cotton moistened with a solution of nitrate of potash, or other substances employed. It could be attached to the skin by paste of gum-arabic, moisture not interfering with the combustion, and when ignited it would cause sufficient counter irritation for most purposes. As to the chemical character of the deflagrating substance I cannot speak with certainty. Can you favor me with a solution of the problen?

I know not, Mr. Editor, whether you will deem these results of sufficient importance to merit a place in your journal, but should you, I would be pleased if you would publish them.

---

*Gunshot wound of the Knee joint.—Recovery.*

As much interest has been excited in the surgical world by attempts at revival of excision of the knee-joint, and all facts bearing upon inflammation of that articulation cannot fail to be of importance, as bearing indirectly upon that question, I have thought that perhaps your readers would feel interested in the details of a case related to me by Dr. Vaughan of this place.

Dr. Hennen, whose opportunities for observation were most ample, and zealously prosecuted, says, in his work entitled the *Principles of Military Surgery*, of injuries of this joint, "in my own practice, I have met with only two cases where the limb was saved after serious gunshot injury, and in one of them only was the perfect use restored." The case in which there was complete restoration of the functions of the joint was that of Lieut. Col. R., — dragoons. The patient was wounded at the battle of Waterloo, the musket-ball entering the outer side of the right knee-joint. During seven days, which included the period of inflammation, the quantity of blood lost both by general and local bleeding, amounted to two hundred and fifty ounces, "while the quantity of food taken during these seven days, or rather the last four, that in comparison of Valsalva's diet was excess." I have cited this case not merely to show the danger attendant upon gunshot injury of the knee-joint, but also because there are many points of similarity in the treatment between it and the one I am about to record.

In the year 1850, Dr. Vaughan was summoned in haste to see Judge G. of Saline county, Mo., who, while hunting, had received an accidental shot from one of his companions. The missile, a large sized buck-shot, entered the knee-joint about midway between the patella and the inner hamstring. Dr. V. saw him first some five or six hours after the receipt of the injury, with Dr. POWELL of Cambridge; he was then suffering considerable pain, and experienced some difficulty in moving his limb. Was ordered a brisk cathartic of sulphate of magnesia, and the continuous application of ice-water dressings, with absolute rest of the limb; as diet he was restricted to the use of three water-crackers per day and three cups of cold water. On the fourth day after the receipt of the injury, traumatic fever supervened, he was copiously bled from the arm, and in the absence of leeches cut cups were freely applied to the neighborhood of the articulation. A solution of tartar emetic was administered to nausea and the saline purgatives repeated. Under this vigorous treatment, the inflammation receded, and the case progressed favorably till the tenth day, when the wife of the patient supposing that Dr. V. really intended to carry out his laughing threat to starve her husband, imprudently allowed him to take a cup of coffee, with a buttered roll and a small piece of steak. The result can be easily foreseen. In a few hours the patient was writhing in agony, the inflammation violently increased in the joint, and the fever re-induced. Ordered a solution of tartar emetic which vomited him freely, and was then continued in nauseating doses for twenty-four hours, cups were also applied. By these means the inflammation was again subdued and he recovered without any further inconvenience than a joint slightly stiffened for a few months, but which recovered its functions completely in a short time.

There are two or three points worthy of note in this case. 1st. That under the rigid treatment, dietetic as well as medicinal, to which this patient was subjected, not the slightest suppuration of the wound appeared, nor did a single abscess form about the joint. 2nd. The beneficial influence exerted upon the wound by the diet, a diet so rigid that truly it could be said of it, "in comparison, the diet of Valsalva was excess;" and the immediate untoward effect exerted by the judges, so far deviating from the directions as to eat a buttered roll and a piece of venison steak not more than three inches square.

GLASGOW, MO., Oct. 29, 1854.

